# Whole-Genome Sequence of an Isogenic Haploid Strain, Saccharomyces cerevisiae IR-2*id*A30(*MAT*a), Established from the Industrial Diploid Strain IR-2

**DOI:** 10.1128/MRA.00018-19

**Published:** 2019-04-25

**Authors:** Kazuhiro E. Fujimori, Yosuke Kobayashi, Taisuke Seike, Takehiko Sahara, Satoru Ohgiya, Yoichi Kamagata

**Affiliations:** a Bioproduction Research Institute (BPRI), National Institute of Advanced Industrial Science and Technology (AIST), Tsukuba, Ibaraki, Japan; b Bioproduction Research Institute (BPRI), National Institute of Advanced Industrial Science and Technology (AIST), Sapporo, Hokkaido, Japan; Broad Institute of MIT and Harvard University

## Abstract

We present the draft genome sequence of an isogenic haploid strain, IR-2*id*A30(*MAT***a**), established from Saccharomyces cerevisiae IR-2. Assembly of long reads and previously obtained contigs from the genome of diploid IR-2 resulted in 50 contigs, and the variations and sequencing errors were corrected by short reads.

## ANNOUNCEMENT

Saccharomyces cerevisiae IR-2 was first isolated in 1985 from fermented food in Indonesia, which showed unusual flocculation properties even under normal cultivation (1–4). In our previous report, we constructed a draft genome sequence of the original IR-2 diploid strain, which comprised ∼300 contigs with a number of heterogenous variations on both chromosomes (5). To overcome these problems, we used consanguineous inbreeding to establish isogenic strain pairs from the IR-2 diploid with improved efficiency in sporulation and spore germination.

IR-2*id*A30 (*MAT***a** and *MAT*α) is a representative isogenic (identical except for the mating-type region) strain pair that we established from the original IR-2 diploid by repetitious syngenesis (zygoses, sporulation, and haploid selection) as follows. To prevent homothallism, two *HO* genes were disrupted by *kanMX* (G418^R^) and *bleMX* (Zeocin^R^) using a traditional lithium acetate (LiAc) transformation method and homologous recombination (6, 7). After four rounds of syngenesis, IR-2*id*A30(*MAT***a** *Δho*::*kanMX*), which exhibited loss of flocculation, was isolated. The antibiotic resistance gene in the *HO* locus was removed by expression of *Cre* recombinase via the pSH47*bla* plasmid (6), in which *URA3* was replaced with the blasticidin S resistance gene *bsd* from the pCAG-Bsd plasmid, resulting in IR-2*id*A30(*MAT***a** *Δho*::*loxP*). Finally, the mating-type region of IR-2*id*A30(*MAT***a**) was converted from *MAT***a** to *MAT*α by *HO* expression via the pHO-*bla* plasmid, in which *Cre* was replaced with *HO*, resulting in IR-2*id*A30(*MAT*α).

We constructed a draft genome sequence of IR-2*id*A30(*MAT***a**) as follows. The cells were grown in yeast extract-peptone-dextrose (YPD) medium (10 g/liter yeast extract, 20 g/liter Bacto peptone, and 20 g/liter d-glucose) at 30°C for 24 hours. Genomic DNA was isolated using the Dr. GenTLE (for yeast) high recovery system. Short reads from IR-2*id*A30(*MAT***a**) were generated on the Ion Personal Genome Machine (PGM) platform using the Ion Xpress Plus fragment library kit. The total number of reads was ∼4.26 million with a mean read length of 378 bases. Approximately 133 million bases of long reads (mean length, 9,643 bases) from IR-2*id*A30(*MAT***a**) were generated using the PacBio RS II sequencer and the PacBio single-molecule real-time (SMRT) kit. Next, the long reads and contigs of the previously obtained IR-2 draft genome sequence (GenBank accession number BAUI00000000) were assembled to create longer contigs using PBJelly2 14.9.9, resulting in 50 contigs totaling 12,013,145 bases. Furthermore, by mapping the short reads using CLC Genomics Workbench 12.0 with the default conditions, 20,176 variations and short indels were corrected (mapping efficiency, 98.72%; coverage, 97.2×). Finally, we obtained 50 contigs of 12,013,960 bases and identified 7,458 potential open reading frames using the CLC Genomics Workbench. A dot plot created using D-GENIES for large genome alignment revealed that 95.61% of IR-2*id*A30(*MAT***a**) bases are highly identical to those of S. cerevisiae S288c (48/50 contigs mapped to S288c chromosomes or mitochondrial sequences) (8). The sequence similarities between S288c and IR-2*id*A30(*MAT***a**) are summarized in Table 1. Interestingly, a large deletion in the IR-2*id*A30(*MAT***a**) genome was observed, corresponding to chromosome I of S288c (approximate positions, 2800 to 11750) (Table 1, contig 19).

**TABLE 1:** Association and mapping rates of the IR-2*id*A30(*MAT***a**) contigs on S288c

Chromosome no.	IR-2*id*A30(*MAT*a) contig no.^a^	Coverage (%)
I	19	83.26
II	6, 24	99.89
III	17	94.54
IV	3, 13	96.26
V	9	97.04
VI	18	96.91
VII	2	95.62
VIII	10	91.35
IX	14, 28	98.56
X	7	93.78
XI	8	95.55
XII	11, 12, 20, 29, 37, 41	96.11
XIII	4	96.25
XIV	15, 16	97.84
XV	1	95.35
XVI	5	92.92

*^a^*Contigs 21, 25 to 27, 30 to 36, 38 to 40, and 42 to 50 mapped to mitochondria (GenBank accession number NC_001224) with redundancy. Contigs 22 and 23 were not mapped on any chromosomes of S288c but included the whole sequence of a 2-micron plasmid (e.g., GenBank accession number CP004554).

Highly accurate genetic information from an isogenic strain with a homogeneous genetic background would provide a useful foundation for developing genetically and metabolically engineered strains for the production of valuable chemical compounds.

### Data availability.

The draft genome sequence of IR-2*id*A30(*MAT***a**) has been deposited in DDBJ under the accession numbers BIMU01000001 to BIMU01000050, DRA accession numbers DRA007804 (Ion PGM) and DRA007805 (PacBio RS II), and BioProject number PRJDB7860.
